# Role of *ACE* I/D polymorphism in pathological assessment of preeclampsia in Pakistan

**DOI:** 10.1002/mgg3.799

**Published:** 2019-06-07

**Authors:** Ghazala Shaheen, Sabahat Sajid, Suhail Razak, Saeeda Batool Mazhar, Tayyaba Afsar, Ali Almajwal, Iftikhar Alam, Sarwat Jahan

**Affiliations:** ^1^ Reproductive Physiology Lab, Department of Animal Sciences Quaid‐i‐Azam University Islamabad Pakistan; ^2^ Community Health Sciences, College of Applied Medical Sciences King Saud University Riyadh KSA; ^3^ Department of Gynae/Obstatrics Pakistan Institute of Medical Sciences Islamabad Pakistan

**Keywords:** angiotensin‐converting enzyme, placenta, preeclampsia, pregnancy, umbilical cord

## Abstract

**Background:**

Preeclampsia (PE) is a pregnancy‐related hypertensive disorder, which may stem from impair placentation. Renin‐angiotensin system is one of the mediators of decidualization and trophoblastic proliferation. In the present study women with PE were studied in a comparison of normotensive controls to determine whether Angiotensin‐converting enzyme (*ACE*) gene I/D polymorphism affect the placental villi and umbilical cord formation with the assessment of biochemical and clinical risk factors.

**Methods:**

Total 400 blood (PE/controls = 200), 400 urine (PE/controls = 200), 90 tissue samples of UC (PE = 50, controls = 40) and 90 placental tissue samples (PE = 50, controls = 40) were recruited. Histomorphological and Histomorphometric analysis were done for placental and umbilical cord tissues. Blood and serum parameters were analyzed, samples were genotyped for I/D polymorphism. Data were statistically analyzed by Independent sample *t*‐test, Chi‐square test and the odds ratio.

**Results:**

Histological study revealed significant increase (*p* < 0.001) in distance from Wharton jelly (in both artery and vein) and outer layer thickness of vein; significant reduction (*p* < 0.01 and *p* < 0.05) in the lumen area of artery and vein. Abnormal villi, more syncytial knots (SK) and a significant decrease in elongated and large villi in PE placentas. Analysis of *ACE* gene determined that genotypic frequencies were statistically significant (*p* < 0.02) among both groups and DD genotype was predominant in the PE group.

**Conclusion:**

Present study reveals that *ACE* I/D polymorphism might affect the normal placental villi and umbilical cord formation in women with PE. In addition, histological studies and genetic evaluation can provide useful information in the determination of various reasons and mechanisms involved in the pathogenesis of PE in Pakistan.

## 1 INTRODUCTION

Preeclampsia is a pregnancy‐related syndrome and is characterized by high blood pressure and onset of proteinuria usually after the 20th week of gestation., while according to revised definition of PE new onset of hypertension in the absence of proteinuria but combined with hematological complications, renal insufficiency, impaired liver function, neurological symptoms also fulfil diagnostic criteria for PE (Kallela et al., 2016). It complicates 3%–8% of pregnancies worldwide resulting in morbidity and mortality at the rate of 10%–15% (Balogun & Sibai, 2017).

Prevalence of preeclampsia is higher in developing countries like Pakistan where PE and eclampsia is around 19% and one in 89 women dies of maternal causes (Naseeb, Bano, & Korejo, 2015; Shamsi et al., 2010). Numerous inclining factors allied to the amplified prevalence of PE are maternal age, pre‐pregnancy obesity, diabetes, multifetal gestations, and chronic hypertension but the main underlying cause of PE remains veiled (Poon, Kametas, Chelemen, Leal, & Nicolaides, 2010). Hence PE seems to have a multifactorial cause and is known as the “disease of theories” (Duckitt & Harrington, 2005; Paré et al., 2014). The direct association between these jeopardy factors and preeclampsia is poorly understood (Clifton, Stark, Osei‐Kumah, & Hodyl, 2012; Steinberg, Khankin, & Karumanchi, 2009). Women having pregnancy complicated by PE had high risk for hypertension, stroke, vascular thromboembolism and ischemic heart diseases. Therefore, preeclampsia raises a red flag concerning the risk of cardiovascular and neurologic disease in later life (Bellamy, Casas, Hingorani, & Williams, 2007).

The most accepted theory about PE describes its two stages; stage 1 is characterized by reduced placental perfusion while stage 2 consists of the multisystem maternal syndrome (Christina et al., 2006). The principal contributor to the pathogenesis of PE is placenta and UC, as the syndrome is resolved after delivery (Raghupathy, 2013; Surbek et al., 2001; Ukah, Magee, Payne, Hutcheon, & von Dadelszen, 2018). In hypertensive pregnancy disorder altered the architecture of the placenta and UC vessel has been observed (Barker, Thornburg, Osmond, Kajantie, & Eriksson, 2010; Ronco, Urrutia, Montenegro, & Llanos, 2009; Sankar, Bhanu, Ramalingam, Kiran, & Ramakrishna, 2013).

Feto‐maternal transport of nutrients, metabolites and respiratory gases can be optimized via successful placentation with the properly developed vascular network (Goyal, Yellon, Longo, & Mata‐Greenwood, 2010). Placental alterations and insufficiency are proposed to be the major contributor toward the onset of PE (Murphy, Smith, Giles, & Clifton, 2006). Normal placentation involves the processes of vasculogenesis and angiogenesis that lead to the formation of a branching network of vessels within the fetal chorionic villi. Renin‐angiotensin system (RAS) displays remarkable changes in multiple organs such as uterus and placenta during pregnancy (Gao, Yallampalli, & Yallampalli, 2012). Additionally, the RAS plays an important role in placental development and regulation of uteroplacental blood circulation. In early pregnancy, angiotensin II, a product of angiotensin‐converting enzyme (ACE) (OMIM 106,180), promotes decidualization and rapid trophoblast proliferation, mainly by binding to its receptor (Hering et al., 2010). Angiotensin II levels are modulated by ACE, whose plasma levels have been associated with the insertion/deletion (I/D) polymorphism in intron 16 of the ACE gene (Hussain, Awan, Gujjar, Hafeez, & Islam, 2018; Mello et al., 2003). Previous studies predicted the marked difference in serum ACE levels in each of the three genotypes (Jackson, Brown, Langdown, Luddington, & Baglin, 2000).

Until now the pathogenecity of PE is poorly understood and further research is required to understand the pathologies of PE. There has been no study analyzing ACE I/D polymorphism with placental and umbilical pathophysiology. Therefore, in the present study we hypothesized that the ACE I/D polymorphism could affect placental and umbilical vasculature leading to susceptibility of preeclampsia in Pakistan.

## 2 MATERIALS AND METHODS

### 2.1 Ethical compliance

The present study was conducted with prior approval from ethical committees of Quaid‐i‐Azam University, Islamabad and collaborating hospitals including Pakistan Institute of Medical Sciences (PIMS), Islamabad and Quaid‐e‐Azam International Hospital, Islamabad. All participants were informed about the study objectives and signed an informed consent.  The study protocol was done in accordance with the principles of the Declaration of Helsinki.

### 2.2 Subjects

In present study, a total of 400 blood (PE = 200, controls = 200), 400 urine (PE = 200, controls = 200) 90 tissue samples of UC (PE = 50, controls = 40) and 90 placental tissue samples (PE = 50, controls = 40) were recruited at Pakistan Institute of Medical Sciences (PIMS) and Quaid‐e‐Azam International Hospital, Islamabad during the period of September 2015 to July 2017. All patients included were <35 years of age. Informed consent and a detailed performa were filled before sample collection.

### 2.3 Inclusion and exclusion criteria

PE was considered with blood pressure ≥140/90 mmHg and new onset of proteinuria. The normotensive control group included women with uncomplicated gestation and blood pressure <125/85mmHg and no proteinuria (Kallela et al., 2016). Exclusion criteria involved diabetes, asthma, kidney disease, hematological disorder, autoimmune disease, urinary tract infection, history of smoking, and eclampsia.

### 2.4 Sampling and storage

Blood and urine samples were collected during the antepartum period before the onset of delivery in labeled tubes for clinical investigations and genetic analysis. UC and Placental tissues were obtained after the termination of pregnancy according to the protocol described by Burton et al., 2014 (Burton et al., 2014).

### 2.5 Biochemical analysis

Complete blood profile was performed on Automated Hematology Analyzer (pocH‐100i, Japan). AMP diagnostic kits (AMEDA labor diagnostic Gmbh, Austria) were used for the determination of plasma creatinine, Alanine aminotransferase (ALT), and aspartate aminotransferase (AST) levels according to manufacturer instructions. Likewise, plasma urea, alkaline phosphatase (ALP), glucose, total bilirubin, and uric acid were determined by Gesan kits (GESAN Production s.r.i, Italy). All the enzyme analysis was done on chemistry analyzer (Piccos 05, Austria). Determination of Sodium, Calcium, and Potassium was done by using Flame Atomic Absorption Spectrophotometer (FAAS) (Varian AA240 FS, USA) while urine analysis was done by Dipstick test using Combur test strips (Roche, Combas®, USA).

### 2.6 Histomorphometric analysis

After fixation in 10% formalin for 48 hr, tissues were subsequently embedded in paraffin wax. Seven‐micrometer thick sections were taken and stained with hematoxylin and eosin (H&E). Slides were observed under the microscope (Leica LB, Germany equipped with canon digital camera, Japan) at different magnifications (20×, 40× and 100×) and were analyzed by using ImageJ 2× software package program. Morphological characteristics of UC and villi were observed and various measurements of UC vessels and villous particles (VP) were taken as determined by Kidron, Vainer, Fisher, & Sharony, 2017 (Kidron et al., 2017).

### 2.7 Molecular analysis

Genomic DNA was extracted by phenol‐chloroform method. The *ACE* (GenBank KJ140509.1) I/D gene was analyzed using polymerase chain reaction (PCR) and primers specified by Devendran et al., 2015 were used (Devendran et al., 2015). The template DNA was amplified by following primers: (forward) 5′‐CTG GAG ACC ACT CCCATC CTT TCT 3′, and (reverse) 5′‐GAT GTG GCC ATCACA TTC GTC AGA T 3′. PCR products (190 bp for the deletion or 490 bp with 287 bp insertion) were detected by electrophoresis on agarose gel containing etheridium bromide. A Gel was visualized for the presence or absence of these fragments using UV trans‐illuminator (Bio‐Rad, UK). After the purification, single band purified product was obtained which was then sequenced using automated ABI PRISM® 3,130 Genetic Analyzer. The sequenced data were analyzed with reference DNA sequence from Ensemble genome browser by Chromas and Sequencher 5.4.6 software.

### 2.8 Statistical analysis

All the data were expressed as a median along with 95% confidence intervals (CI). Quantitative data were expressed as mean ± *SE*. Hardy–Weinberg equilibrium was tested for genotypic distribution of *ACE* I/D polymorphism. Odds Ratios (OR) were calculated as a measure of the degree of relative risk for PE. Significant difference between groups for clinical, demographic data and genotypic results was determined by applying Independent sample *t* test and Chi‐squared test (χ^2^). Association of *ACE* I/D polymorphism with anthropometric parameters of study subjects was checked by One‐Way analysis of variance (ANOVA) followed by post hoc Dunnett's test using IBM SPSS Statistics 21 software.

## 3 RESULTS

**Figure 1:**
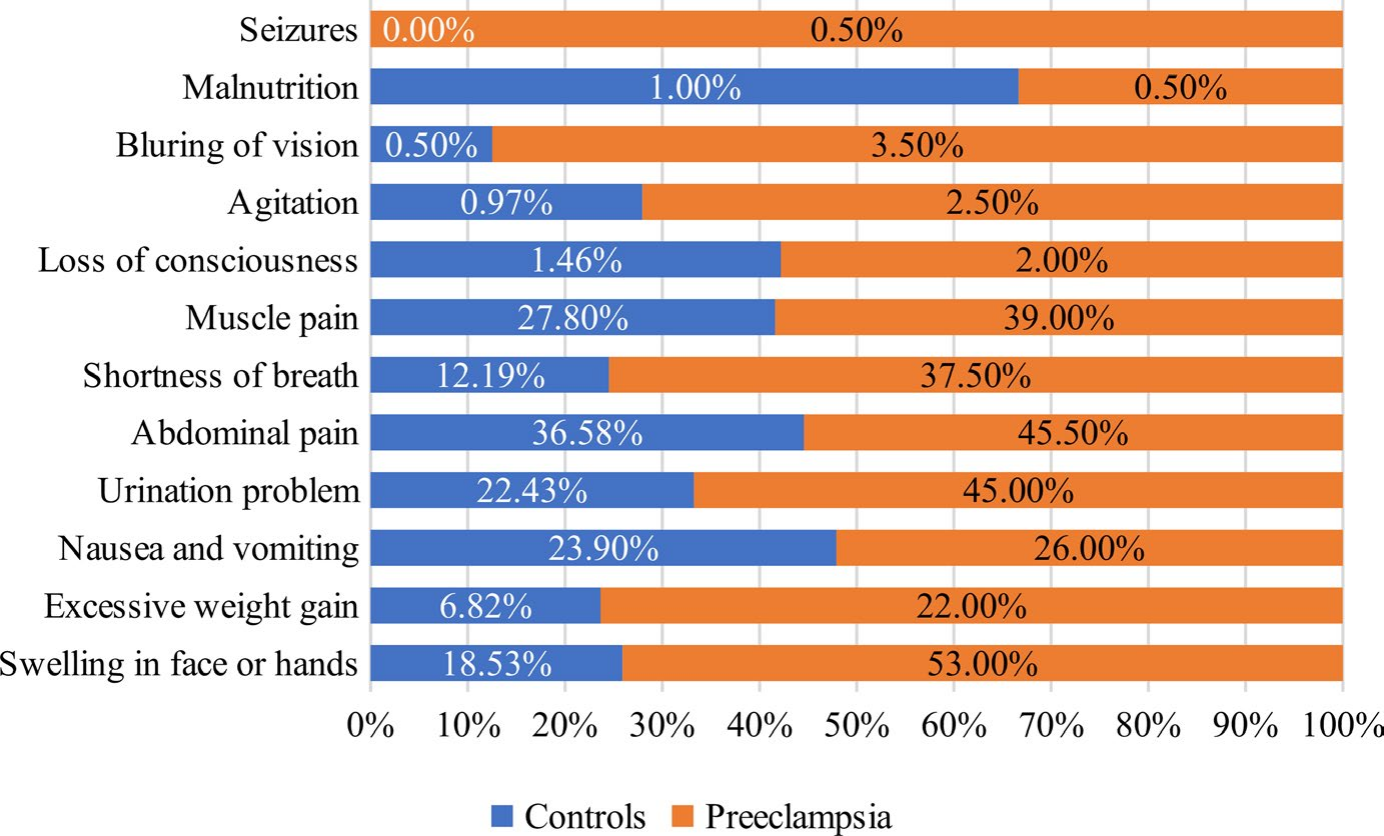
Pregnancy history of control group and preeclamptic group

Demographic characteristics have been summarized in Table 1, where significant difference in age (*p* = 0.002), BMI (*p* = 0.047), gestational age (*p *≤ 0.001) and blood pressure (*p *≤ 0.001) among both groups is evident. Although case patients have 3 weeks lesser pregnancy period, weight gain was higher in case patients as compared to control group. Similarly, a significant difference was observed in infants’ weight (*p* < 0.001) and urine proteins (*p* < 0.001) of mothers with PE and normotensive mothers. The findings of present study showed that pregnancy and medical history revealed expected results—as case patients were more likely to be suffering from pregnancy‐associated symptoms as compared to control group. A large proportion of case patients had a history of preeclampsia in previous pregnancies as well as in the inheritance (Figure 1).

**Table 1:** Characteristics of the study population

Parameters	Controls	PE Patients	*p* value
Age (Years)	27.017 ± 0.32	28.52 ± 0.34	0.002
BMI (kg/m^2^)	27.67 ± 0.28	28.59 ± 0.360	0.047
Gestational age (Weeks)	36.32 ± 0.36	33.12 ± 0.15	<0.001
Systolic blood pleasure (mmHg)	114.71 ± 0.51	154.36 ± 1.54	<0.001
Diastolic blood pleasure (mmHg)	73.88 ± 0.45	100.95 ± 1.12	<0.001
Abortions	0.51 ± 0.064	0.71 ± 0.033	0.09
Infants’ weight (kg)	3.03 ± 0.03	2.41 ± 0.021	<0.001

### 3.1 Biochemical analysis

Biochemical investigations revealed a significant increase in TLC (*p* = 0.028) and hematocrit (*p* = 0.016) among preeclamptic subjects as compared to the control group while a non‐significant change was observed in other blood parameters among these two groups. Significantly, elevated (*p* < 0.001) levels of alkaline phosphatase, serum urea, uric acid, and urine proteins were reported in PE. While, total bilirubin (*p* = 0.019), AST (*p* = 0.012), serum calcium (*p* = 0.002), and sodium (*p* = 0.010) concentrations were reduced in case patients as compared to control group (Tables 2 and 3).

**Table 2:** Levels of various blood parameters in normotensive group and preeclamptic group

Parameters	Controls	PE Patients	*p* value
TLC (10^9^/L)	10.03 ± 0.17	11.43 ± 0.36	0.028
Hemoglobin (g/dl)	11.88 ± 0.76	11.29 ± 0.101	0.445
WBC (10^9^/L)	10.41 ± 0.31	9.00 ± 0.28	0.232
RBC (10^12^/L)	4.18 ± 0.046	4.21 ± 0.038	0.725
Platelets (10^9^/L)	259.41 ± 5.53	242.86 ± 6.58	0.081
Hematocrit (%)	32.85 ± 0.38	35.45 ± 0.25	0.016
PCV/HCT	36.06 ± 0.90	34.66 ± 0.33	0.202
MCV (fL)	80.39 ± 1.053	82.90 ± 0.90	0.093
MCH (pg)	26.64 ± 0.29	27.08 ± 0.30	0.327
MCHC (g/dl)	32.39 ± 0.20	32.03 ± 0.14	0.171
RDW‐CV (%)	16.39 ± 0.46	15.97 ± 0.23	0.417
Neutrophils (%)	69.20 ± 1.03	68.45 ± 1.35	0.881
Lymphocytes (%)	21.76 ± 0.58	21.21 ± 0.66	0.571

**Table 3:** Blood biochemistry and urine examination of healthy women and preeclamptic women

Parameters	Controls	PE Patients	*p* value
Blood sugar random (mg/dL)	86.56 ± 1.20	90.22 ± 1.41	0.071
Alkaline phosphatase (U/L)	95.77 ± 4.47	147.33 ± 4.92	<0.001
Bililubin total (mg/dl)	0.39 ± 0.03	0.29 ± 0.01	0.019
Serum creatinine (mg/dL)	0.77 ± 0.01	0.81 ± 0.01	0.128
Serum urea (mg/Dl)	17.08 ± 0.56	21.25 ± 0.74	<0.001
SGPT (ALT) (U/L)	20.50 ± 2.29	21.94 ± 1.45	0.068
SGOT (AST) (U/L)	21.63 ± 0.64	30.44 ± 1.78	0.012
Uric acid (mg/dL)	3.53 ± 0.08	5.67 ± 0.18	<0.001
Calcium (nmol/l)	1.87 ± 0.05	1.26 ± 0.01	0.002
Potassium (nmol/l)	3.97 ± 0.02	3.67 ± 0.05	0.152
Sodium (nmol/l)	138.8 ± 0.17	134.5 ± 0.44	0.010
Sp. gravity	1.01 ± 0.00	1.01 ± 0.00	0.373
Proteins	0.08 ± 0.03	2.29 ± 0.05	<0.001
PH	6.10 ± 0.06	5.96 ± 0.04	0.143
Urobilinogen	0.22 ± 0.03	0.22 ± 0.01	0.747

### 3.2 Histological analysis

**Figure 2:**
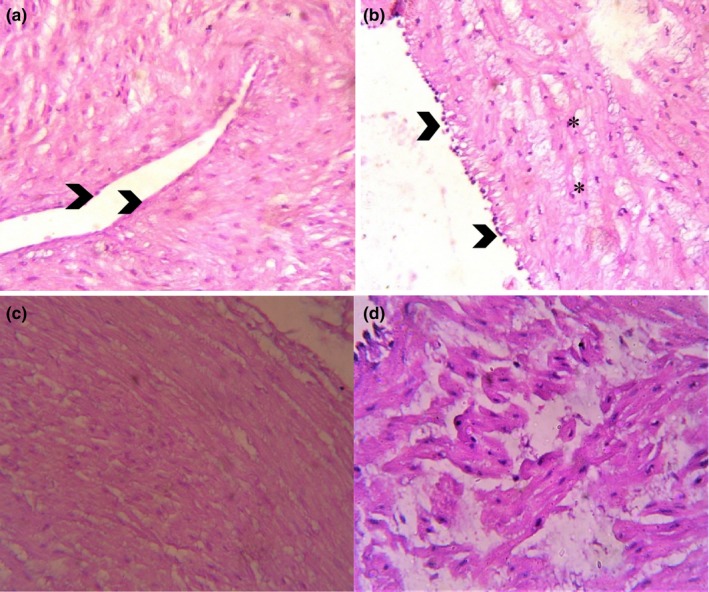
(a) Endothelium of umbilical artery appear with flattened pale stained nuclei (arrowheads), resting on subendothelial connective tissue in control group. (b) Endothelium of a PE female umbilical vein (UV) with darkly stained nuclei (arrowheads) and infiltrations of subendothelial smooth muscle cells (*). (c) Nuclei arranged in a circular manner in the vessel wall of umbilical artery. (d) Nuclei dispersed in a disorganized manner in vessel wall of umbilical artery. (40×)

**Figure 3:**
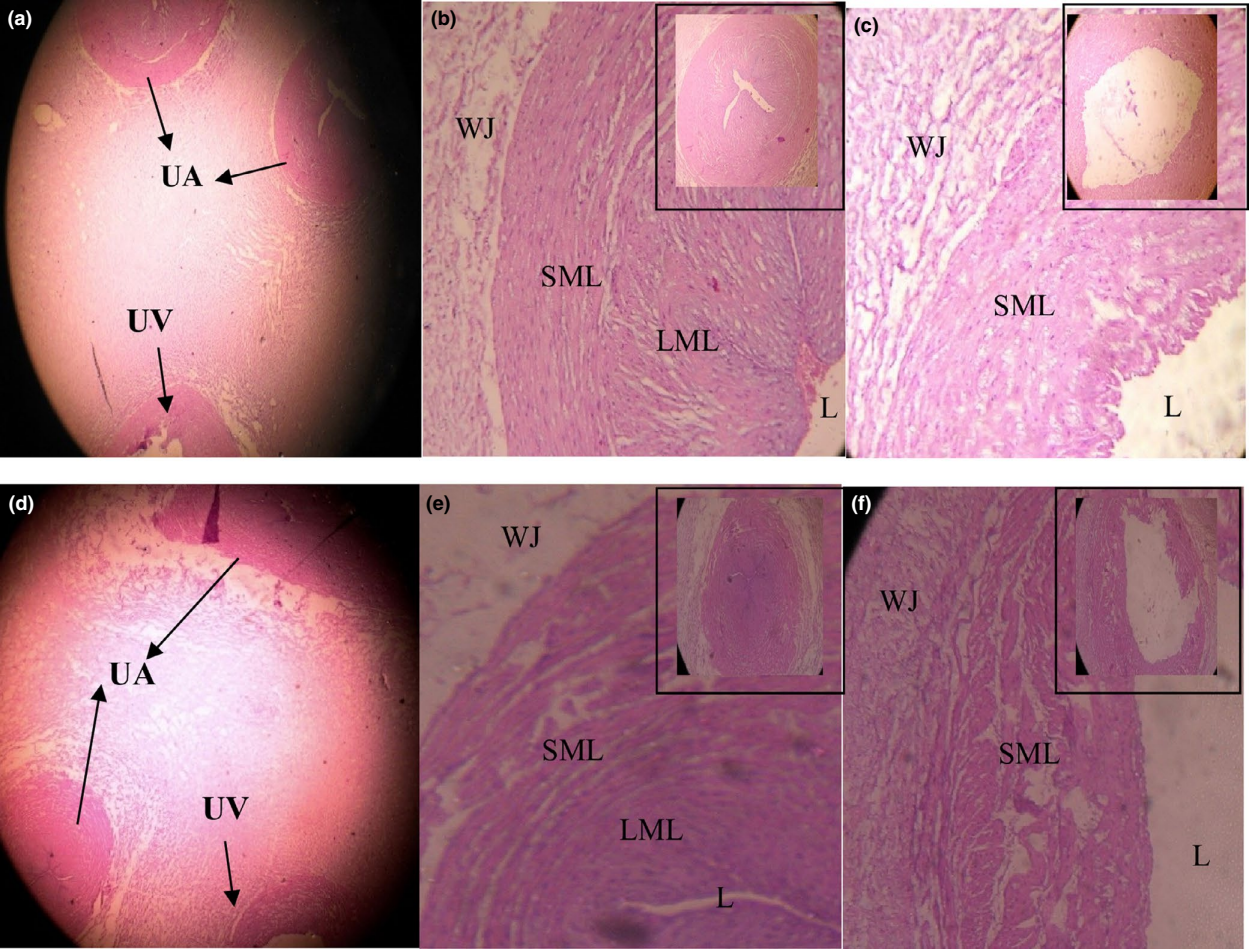
(a, b, and c) Umbilical cord vessels of a normal pregnant woman. (d, e and f). Umbilical cord vessels of a preeclamptic female: Umbilical arteries (UA), Umbilical vein (UV), Wharton's jelly (WJ), outer smooth muscle layer (SML), inner longitudinal muscle layer (LML) and lumen (L). (4×, 10×, 40×)

Results of histomorphological findings indicated endothelium of a PE female umbilical vein (UV) with darkly stained nuclei (arrowheads) and infiltrations of subendothelial smooth muscle cells as compared to control group (Figure 2). Histomorphometric analysis of UC revealed that distance from Wharton's jelly in arteries showed a significant increase (*p* < 0.001) while, significant reduction (*p* < 0.01) in lumen area was observed in preeclamptic women.. A non‐significant change was observed in thickness of outer and inner layer. In the vein, distance from Wharton's jelly and layer thickness revealed significant (*p* < 0.001) increase while, lumen area showed a significant (*p* < 0.05) decline when compared with the control. (Figure 3, Table 4).

**Table 4:** Histomorphometric parameters of umbilical cord vessels in control and preeclamptic group

Parameters	Groups	
	Control	PE
Artery		
Distance from Wharton jelly (µm)	10.30 ± 0.51	20.60 ± 1.10***
Outer layer thickness (µm)	97.11 ± 1.89	113.71 ± 2.27
Inner layer thickness (µm)	78.20 ± 1.97	93.60 ± 2.19
Lumen area (µm)	15.02 ± 0.51	10.22 ± 0.78**
Vein		
Distance from Wharton jelly (µm)	6.30 ± 0.29	14.82 ± 1.22***
Layer thickness (µm)	109.71 ± 2.20	153.41 ± 5.17***
Lumen area (µm)	194.53 ± 4.45	158.40 ± 6.66*

Note

Level of Significance:

* *p* < 0.05.

** *p* < 0.01.

*** *p* < 0.001.

**Figure 4:**
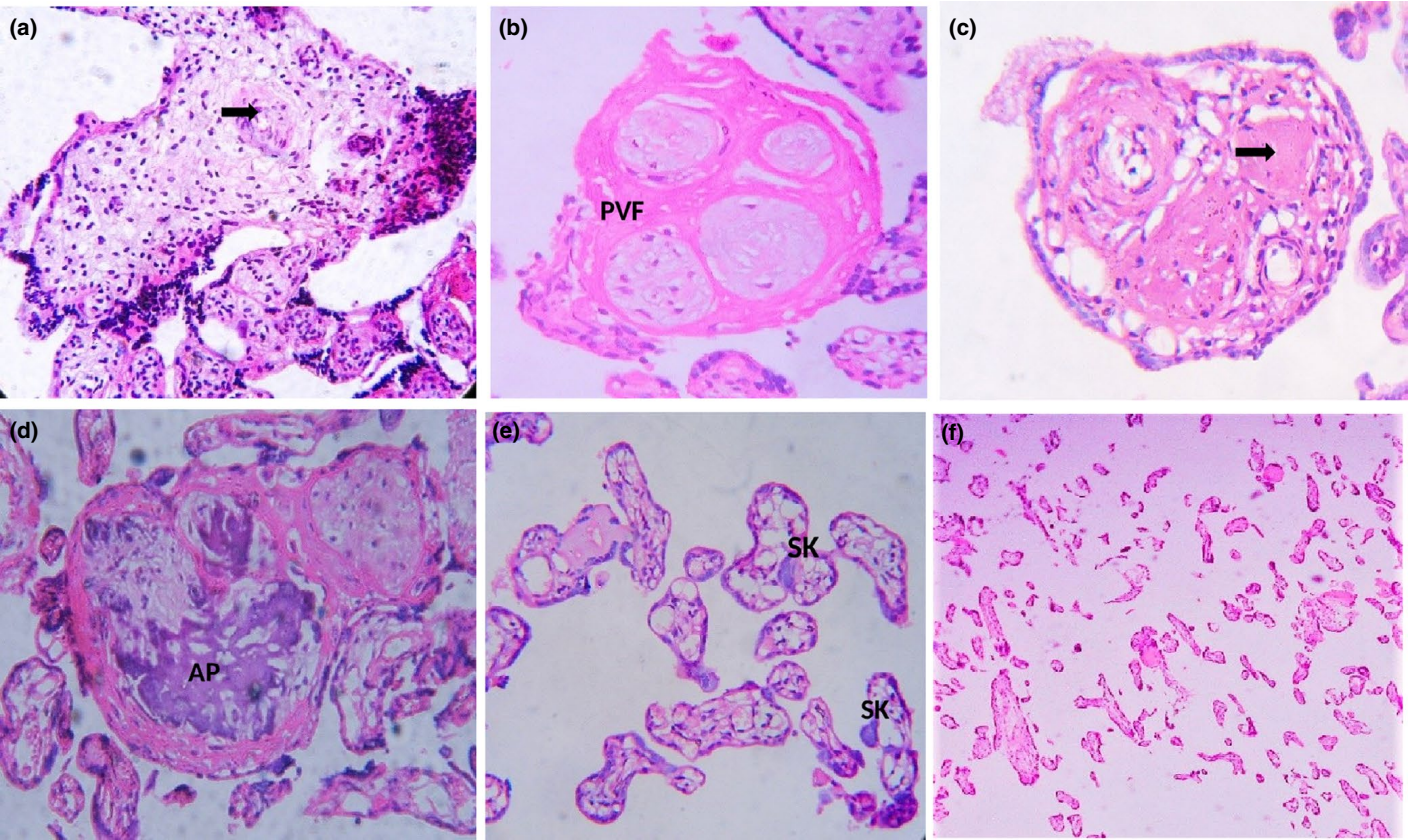
Hematoxylin and eosin stained sections of PE placentas. (a) Stem villous having contracted vessels. (b) Perivillous fibrin (PVF) deposition in stem villous. (c) Obliteration of vascular lumen in stem villous. (d) Atheromatous plaque (AP) formation in stem villous (e) syncytial knots (SK). (f) Thin and elongated villi in PE. (a–e, 40×, f, 20×)

Histomorphological findings of PE placentas showed Stem villous with contracted vessels (Figure 4a), perivillous fibrin (PVF) deposition (Figure 4b), obliteration of vascular lumen (Figure 4c) and atheromatous plaque (AP) (Figure 4d). Similarly, syncytial knots (SK) (aggregated syncytial nuclei in the outer surface placental villous) along with thin and elongated villi were more in PE placentas than control group (Figure 4e & f).

**Figure 5:**
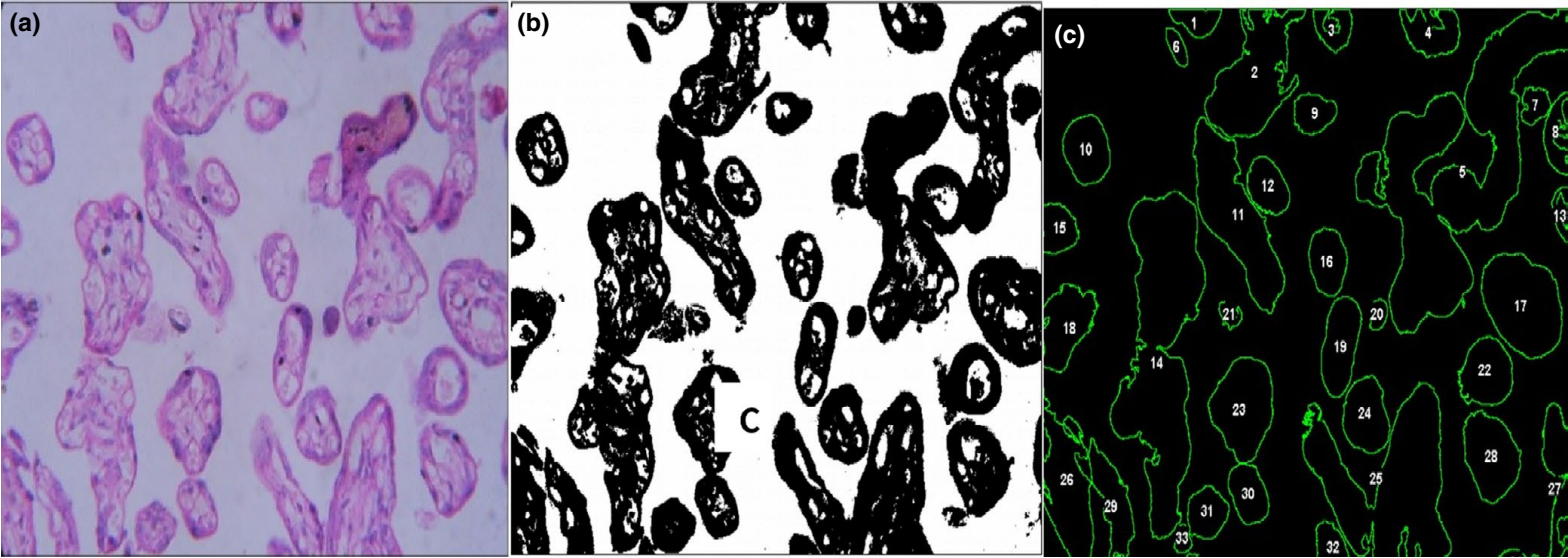
Analysis of placental villi by ImageJ software. (a) Normal appearance of villi (H&E, 40 X). (b) Villi with adjusted threshold. (c) Outlines of villi after particle analysis

The histomorphometric analysis of placenta revealed a remarkable change in VP physical characteristics VP were further divided into three types small, elongated and large according to the criteria given by Kidron et al., 2017. Small VP was rounded with greater than 0.3 circularity (i.e., VP 16, 19, 22, 23, Figure 5e), elongated VP have a circularity of 0.2–0.3 (VP 14, Figure 5e) and large VP were determined by irregular outlines with less than 0.2 circularity (VP 5, Figure 5e). A significant decrease in the %age area of small VP was reported in PE placentas and approximately 47% area was occupied by small VP. In addition, other parameters including area, perimeter, circularity, ferret, and minimum ferret diameter were reduced in elongated and large VP of PE placentas as compared to the control group (Table 5).

**Table 5:** Measurements of villous particles conferring to size in normotensives and preeclamptic group

Size	Parameters	Controls	PE
Small	No. of villi (%)	922 (44.32)	1,390 (46.83)
	Area (×10^4^ µm)	1.41 ± 0.20	1.11 ± 0.23
	Perimeter (×10^2^ µm)	10.00 ± 0.21	9.43 ± 0.23
	Circularity	0.50 ± 0.005	0.4985 ± 0.005
	Feret diameter (µm)	147.71 ± 4.58	112.92 ± 5.22
	Minimum ferret diameter (µm)	148.66 ± 2.001	145.83 ± 2.71
	% area per image	88.01 ± 0.38	84.43 ± 0.62***
Elongated	No. of villi (%)	713 (34.27)	845 (28.47)
	Area (×10^4^ µm)	7.88 ± 0.79	5.74 ± 0.48*
	Perimeter (×10^2^ µm)	16.60 ± 0.78	14.32 ± 0.63*
	Circularity	0.24 ± 0.002	0.23 ± 0.001***
	Feret diameter (µm)	400.73 ± 21.26	362.65 ± 16.003*
	Minimum ferret diameter (µm)	223.66 ± 10.54	185.24 ± 8.62**
	% area per image	89.45 ± 0.71	89.24 ± 0.85
Large	No. of villi (%)	445 (21.39)	733 (24.69)
	Area (×10^4^ µm)	13.80 ± 1.04	11.65 ± 1.09
	Perimeter (×10^2^ µm)	38.92 ± 2.14	33.27 ± 1.28*
	Circularity	0.11±0.002	0.1 ± 0.001
	Feret diameter (µm)	593.70 ± 20.40	459.85 ± 12.58***
	Minimum ferret diameter (µm)	308.19 ± 12.21	237.83 ± 7.66
	% area per image	89.13 ± 0.40	90.66±0.0.29

Note

Level of Significance:

* *p* < 0.05.

** *p* < 0.01.

*** *p* < 0.001.

### 3.3 Molecular analysis

The genotypic frequency for *ACE* I/D polymorphism differed significantly between the PE and control groups while the allelic frequency showed a non‐significant difference between both groups (Table 7). The frequencies of DD, ID and II genotypes among PE patients were 52%, 43%, and 5% versus 64%, 21%, and 15.15% for the control group, respectively. DD genotype was found prevalent in the PE group than in the control group. The genotype DD versus ID showed strong association (OR: 0.406; CI: 0.779–0.920; ϰ^2^: 4.766; *p*: 0.02) with the PE group (Table 6). After sequencing, data were analyzed and no change was observed in the nucleotide sequence of PE and control group. Both groups were further divided into three groups based on genotypes which were DD, ID, and II. Association of *ACE* polymorphism was checked, and the significant association of DD genotype with a systolic blood pressure of PE patients and gestational age in both groups (Table 7).

**Table 6:** Genotypic and allelic frequencies of ACE gene I/D polymorphism in the study groups

	Genotype	Control (*n* = 200)	PE (*n* = 200)	*p* value
Genotypic frequency	DD	104 (52%)	128 (64%)	0.02
	ID	86 (43%)	42 (21%)	
	II	10 (5%)	30 (15.15%)	
Allelic frequency	I	0.268	0.257	0.571
	D	0.731	0.742	

ACE Genotypes	Control versus PE			*p* value
	Odds ratio	CI (95%)	ϰ^2^	
DD versus II	2.22	0.56–8.79	1.342	0.20
DD versus ID	0.406	0.779–0.920	4.766	0.02
DD versus ID + II	0.615	0.296–1.280	1.697	0.132

**Table 7:** Association of *ACE* (I/D) polymorphism with anthropometric parameters of study subjects

Parameters	Groups	*ACE* (I/D) Polymorphism			*p* value
		DD	ID	II	
Age (Years)	Control	26.51 ± 0.47	27.73 ± 0.45	26.1 ± 0.97	0.15
	Patient	28.66 ± 0.46	28.60 ± 0.61	27.93 ± 0.89	0.75
BMI (kg/m^2^)	Control	28.21 ± 0.42	27.37 ± 0.39	23.25 ± 0.50	0.21
	Patient	28.28 ± 0.43	29.53 ± 0.82	28.60 ± 0.91	0.40
Gestational Age (Weeks)	Control	34.96 ± 0.54	38.26 ± 0.44	39.78 ± 0.76	<0.001
	Patient	30.92 ± 0.44	33.52 ± 0.18	35.53 ± 0.09	<0.0001
Systolic Blood Pleasure (mmHg)	Control	113.1 ± 0.57	116.50 ± 0.88	116 ± 2.11	0.15
	Patient	151.89 ± 1.88	163.18 ± 3.27	156.5 ± 4.45	0.03
Diastolic Blood Pleasure (mmHg)	Control	73.6 ± 0.51	75.54 ± 0.80	76 ± 1.56	0.09
	Patient	99.71 ± 1.36	104.54 ± 2.17	101.81 ± 3.34	0.285

## 4 DISCUSSION

Placental formation ensures intimate connection between maternal and fetal blood and nutrients circulation (Goyal et al., 2011). PE is often related to poor placentation as its symptoms resolves after delivery by the removal of placenta. RAS has an important role in the placental formation and regulation of uteroplacental blood circulation (Gao et al., 2012). ACE (a component of RAS) promotes decidualization and trophoblastic proliferation by binding to its receptor. The current study provides the evidence of poor placentation and UC development due to ACE I/D polymorphism.

According to current findings, damage in hepatic and renal system was observed by a boost in standard blood markers such as ALP, Total bilirubin, AST, urea, proteins and uric acid. Serum electrolytes including calcium and sodium levels decreased significantly in preeclamptic group as compared to control. Decreased level of sodium retention increased sensitivity to angiotensin which leads to hypertension, edema, and proteinuria, the diagnostic triad of preeclampsia (Anuradha & Shamshad, 2016).

Abnormalities in UC and placental villi have been observed in PE patients. Lumen area of the umbilical artery, vein, and distance of WJ in vein showed a significant reduction in women as compared to control. These results in an agreement to the previous studies (Barnwal, Rathi, Chhabra, & Nanda, 2012). Wall thickness of vein shows a significant rise in PE when compared with control as observed earlier (Inan et al., 2002; Yasoob, Bokhari, & Bukhari, 2014). According to histological perspectives of placenta, abnormalities in stem villi were detected. Preceding studies also exhibited such results in which contracted vessels, PVF deposition, and obliteration of vascular lumen AP formation in stem villous was observed in PE placentas (Allaire, Ballenger, Wells, McMahon, & Lessey, 2000; Khong et al., 2016; Sankar et al., 2013).

In present observations SK was more in PE patients as shown in the study conducted by Sankar et al. (2013) proposing that the functional impairment of the placenta is due to structural alterations of the villous syncytiotrophoblast (Sankar et al., 2013). Thin and elongated villi were also more in PE as detected by Khong et al., 2016 (Khong et al., 2016). Placental villi represented a significant decrease in area and circularity of PE as compared to controls. The difference was more prominent in elongated VP (150–250 µm in diameter) which are probably similar to intermediate villi, and large villi (>250 µm in smaller diameter) representing aggregates of adherent or closely approximated villi or immature intermediate villi. Terminal villi were characterized as small VP (50–150 µm in diameter) showing no significant difference in both groups. Maternal and fetal circulation takes place through these villi, in case of poor vascularization the fetus is at high risk of hypoxia and low birth weight (Krielessi et al., 2012; Teasdale, 1987). All these abnormalities arise in PE patients due to poor placentation and vascularization which is the key reason of etiology of PE (Mello et al., 2003). The possible reason of these alterations is the defect in RAS manly *ACE* gene function.

In our study *ACE* I/D genotype distribution, DD, ID, and II in PE group differed significantly from the control group. DD genotype is found prevalent as compared to other genotypes (ID, II) among both study groups, and is predominant in PE group (64%) than the controls (52%) with a significant association with systolic blood pressure. It could be suggested that DD genotype increases *ACE* circulatory levels as compared to other genotypes leading to hyper‐production of AngII, which increases oxidative stress, vasoconstriction and activate other mechanisms which lead to impair placentation and abnormal development of uteroplacental circulation (Mello et al., 2003). Above mentioned abnormalities of placental villi and UC may result from the *ACE* I/D polymorphism detected in PE patients in the present study. Association between D allele frequency and preeclampsia has been suggested previously in many different populations (Aung, Konoshita, Moodley, & Gathiram, 2018; Zhong, Wang, Zhu, & Zhao, 2012). Thus, there is also region distribution in an analysis of the ACE gene polymorphism.

## 5 CONCLUSION

Findings of this study contributed to the histomorphological and histomorphometric characteristics of placental and UC tissues, allowing for a greater agreement that pathological examination of the placenta and UC are an important parameter for detection of their alterations during PE. Further work must be done on histological examination of the placenta and UC which can provide useful information in the determination of various reasons and mechanisms involved in poor pregnancy outcomes and useful to health care providers both for parent counseling and as a legal defense in cases of medical malpractice allegations. In addition, our investigations suggest that I/D polymorphism of the *ACE* gene might increase the risk to develop PE in Pakistani women. Due to small population size and selection of a little portion of the *ACE* gene, it is necessary to conduct large sample sized studies in the future to elucidate the association of the *ACE* gene with the severity of the PE. This approach will allow close vigilance and easy referral for pregnant women at risk that will subsequently improve maternal and child health in the Pakistani population.

## 6 DECLARATION OF INTEREST

The authors report no declarations of interest.

## AUTHOR CONTRIBUTION

GS designed the study, conceived the study and analyzed the results. SR, AA, TA, SJ, and SS conceived an initial part of the study, performed the experiment, histology and helped in compiling the results. GS and SS performed experiment. GS, SM, IA, and SR helped in writing the results. IA and SR wrote the paper with input from all other authors SJ, SR, TA, and AA made substantial contribution in interpretation of data and revising the manuscript for intellectual content. All authors read and approved the final manuscript.
